# Development of a model to predict vestibular schwannoma growth: An opportunity to introduce new wait and scan strategies

**DOI:** 10.1111/coa.13661

**Published:** 2020-11-06

**Authors:** Mayke A. Hentschel, Gerjon Hannink, Stefan C. A. Steens, Jef J. S. Mulder, Maroeska M. Rovers, Henricus P. M. Kunst

**Affiliations:** ^1^ Department of Otolaryngology Radboud Institute for Health Sciences Radboud university medical center Nijmegen The Netherlands; ^2^ Department of Operating Rooms Radboud Institute for Health Sciences Radboud university medical center Nijmegen The Netherlands; ^3^ Department of Radiology and Nuclear Medicine Radboud university medical center Nijmegen The Netherlands; ^4^ Department of Health Evidence Radboud Institute for Health Sciences Radboud university medical center Nijmegen The Netherlands; ^5^ Department of Otolaryngology Maastricht UMC+ Maastricht The Netherlands

**Keywords:** growth, prediction model, vestibular schwannoma, wait and scan

## Abstract

**Objectives:**

To develop a prediction model to predict vestibular schwannoma (VS) growth for patients in a wait and scan (W&S) strategy.

**Design:**

Retrospective cohort study.

**Setting:**

Tertiary hospital (Radboud university medical center, Nijmegen, the Netherlands).

**Participants:**

Patients with unilateral VS, entering a W&S strategy and at least one follow‐up MRI available. Data on demographics, symptoms, audiometry and MRI characteristics at time of diagnosis were collected from medical records.

**Main outcome measures:**

Following multiple imputation, a multivariable Cox regression model was used to select variables, using VS growth (≥2 mm) as outcome. Decision curve analyses (DCA) were performed to compare the model to the current strategy.

**Results:**

Of 1217 analysed VS patients, 653 (53.7%) showed growth during follow‐up. Balance complaints (HR 1.57 (95% CI: 1.31‐1.88)) and tinnitus complaints in the affected ear (HR 1.36 (95% CI: 1.15‐1.61)), Koos grade (Koos 1 is reference, Koos 2 HR 1.03 (95% CI: 0.80‐1.31), Koos 3 HR 1.55 (95% CI: 1.16‐2.06), Koos 4 HR 2.18 (95% CI: 1.60‐2.96)), time since onset of symptoms (IQR HR 0.83 (95% CI: 0.77‐0.88) and intrameatal diameter on MRI (IQR HR 1.67 (95% CI: 1.42‐1.96)) were selected as significant predictors. The model's discrimination (Harrell's C) was 0.69 (95% CI: 0.67‐0.71), and calibration was good. DCA showed that the model has a higher net benefit than the current strategy for probabilities of VS growth of >12%, 15% and 21% for the first consecutive 3 years, respectively.

**Conclusions:**

Patients with balance and tinnitus complaints, a higher Koos grade, short duration of symptoms and a larger intrameatal diameter at time of diagnosis have a higher probability of future VS growth. After external validation, this model may be used to inform patients about their prognosis, individualise the W&S strategy and improve (cost‐)effectiveness.

**Key points**

- Patients with balance and tinnitus complaints, a higher Koos grade, short duration of symptoms and a larger intrameatal diameter at time of diagnosis have a higher probability of future VS growth.
- After external validation, this model may be used to inform patients about their prognosis, individualise the W&S strategy and improve (cost‐)effectiveness.

## 1 INTRODUCTION

Over the past years, conservative management of unilateral vestibular schwannoma (VS) has gained popularity.^1^ Currently, a “wait and scan” (W&S) strategy is preferred in the majority of patients with a newly diagnosed VS.^2^ The aim of a W&S strategy is to detect VS growth by means of repeated magnetic resonance imaging (MRI) examinations. In case of a large VS compressing surrounding tissues and/or detected growth during W&S, patients are usually referred for treatment, consisting of radiation therapy (ie stereotactic radiosurgery [eg Gamma Knife] or fractionated radiotherapy), or microsurgery. A large proportion of VSs observed within a W&S strategy remains stable in size and thus remains untreated during life.^3^, ^4^ VSs are usually diagnosed in the sixth decade of life.^2^, ^5^ W&S strategies are known to vary. A survey among otolaryngologists revealed several strategies, consisting of MRIs every 1‐5 years, either continued until a specific age (75 or 80), for a specific period (4‐21 years) or lifelong.^6^ Thus, patients undergo a large number of MRIs during a lifetime. This contributes to the high costs associated with VS care and burdening of hospital visits for patients.^7^ Preferably, we would select patients that need to be monitored carefully, because their VS has a high risk of future growth (and thus treatment), while others can be monitored less strictly or may even be omitted from further controls. This might improve (cost‐)effectiveness of the W&S strategy, contribute to individualised patient care and result in better informed patients regarding the prognosis of their disease due to improved patient counselling. Therefore, the purpose of this study was to develop a clinical prediction model that can be used to predict VS growth for newly diagnosed patients assigned to a W&S strategy.

## 2 MATERIALS AND METHODS

We developed a multivariable prediction model to predict VS growth. Information on potential predictors and the outcome was retrospectively collected from patient records. The study protocol was published online (in Dutch, summary in English: https://www.zonmw.nl/nl/onderzoek-resultaten/doelmatigheidsonderzoek/programmas/project-detail/doelmatigheidsonderzoek/cost-effective-diagnostic-strategies-in-patients-with-asymmetrical-hearing-impairment-or-unilateral/verslagen/). The study was reported following the Transparent Reporting of a multivariable prediction model for Individual Prognosis Or Diagnosis (TRIPOD) statement.^8^

### 2.1 Study population

Most patients with a newly diagnosed VS are referred to a specialised tertiary centre to determine further management. We consulted medical records of all patients that got assigned the diagnostic code “cerebellopontine angle (CPA) lesion” and/or had undergone an MRI of the CPA in a tertiary hospital between 1990 and July 2016. We identified patients with a unilateral VS diagnosed by means of MRI. All patients initially assigned to a W&S strategy were included. The local W&S strategy prescribes MRIs at 1, 2, 3, 5, 7, 9, 12 and 15 years following diagnosis, then continuing every 5 years during the remaining lifetime of a patient. The W&S strategy could either be carried out in our own institution or in the referring clinic. To be able to study VS growth at least one follow‐up MRI (either images or a report) had to be available. Thus, patients diagnosed with other modalities than MRI, those with bilateral VSs (ie neurofibromatosis), VSs that immediately had been treated, or without available follow‐up, or CPA lesions other than VS were excluded.

### 2.2 Outcome

#### 2.2.1 VS growth

Time‐to‐VS growth was defined as the number of months between the baseline MRI and the one on which VS growth was detected.

MRI examinations were assessed by one of the authors [MH] to determine whether growth had occurred. Each MRI was compared to the baseline MRI. Largest VS diameter was measured in two directions on axial images, that is parallel to the internal auditory canal (split in an intra‐ and extrameatal portion delineated by the petrous bone)^9^ and largest extrameatal diameter parallel to the petrous bone. All measurements were rounded off to millimetres. Contrast enhanced T1‐weighted images were preferably used to assess lesions. In case these were unavailable, T2‐weighted images were used.

For intrameatal VSs, an increase in tumour diameter ≥2 mm parallel to the internal auditory canal was considered growth. For extrameatal VSs, growth was considered an increase ≥2 mm of the extrameatal portion in either direction.^9^

Whenever the W&S strategy was performed in another hospital and baseline or follow‐up MRI images were unavailable, we evaluated growth based on the radiologists’ reports. When the report stated that growth had occurred, we assumed this to be true.

### 2.3 Potential predictors

Twenty‐two potential predictors were selected based on literature and interviews with three experts (otolaryngologists from our centre, working in the field of VS). Demographics (sex [male/female] and age), symptoms, pure‐tone audiometry (PTA) and MRI findings at time of diagnosis were collected from the patients’ medical records. Presence of complaints of hearing loss, tinnitus and aural pressure on the affected side were collected [yes/no]. The onset of hearing loss was classified [sudden/gradual]. Also, the presence of vertigo or balance complaints was collected [present/absent]. The time since onset of symptoms up to diagnosis was expressed in months [continuous variable].

#### 2.3.1 Pure‐tone audiometry

PTA data were retrieved from the clinical audiology database system AudiologicX (version 1.0.6, MarYor, the Netherlands). In our centre, PTA is performed in a soundproof room according to standard audiometric protocols. We collected hearing thresholds in dB hearing loss of octave frequencies 0.5, 1, 2, 4 and 8 kHz for air conduction (AC). Measurements on the affected side were used. Results of PTA performed within a range of six months prior and after diagnosis were included. In case a patient had multiple PTA examinations available, the one most proximate to the diagnostic MRI was selected.

#### 2.3.2 Baseline MRI

Baseline MRI images were assessed to determine VS size [continuous variable, in mm] as previously described, aspect [homogeneous/inhomogeneous], presence of cysts within the inhomogeneous VSs [yes/no] and Koos grading scale [grade 1‐4], representing the lesion's size in relation to surrounding structures.^10^

### 2.4 Data analysis

Descriptive statistics were used to summarise the data. For 15 of the 22 potential predictors, data were missing, ranging between 2.2% and 63.8% (Table 1). We assumed missing data to be missing at random (MAR). Imputation of missing values was performed using multiple imputation by chained equations, creating 25 imputation sets.^11^

**Table 1:** Characteristics of included patients with a unilateral VS obtained in a W&S strategy

At time of diagnosis	Descriptives n (%)^a^					
	Total N = 1217	Missing	VS growth n = 653	Missing	No VS growth n = 564	Missing
Male gender	632 (51.9)	—	334 (51.1)	—	298 (52.8)	—
Age (y, median [range])	58.5 (16.5‐88.0)	—	57.7 (16.5‐88.0)	—	59.2 (23.9‐87.9)	—
Symptoms						
Hearing loss^b^	1105 (90.8)	—	598 (91.6)	—	507 (89.9)	—
Onset of hearing loss		776 (63.8)		418 (64.0)		358 (63.5)
Sudden	104 (8.5)		57 (8.7)		47 (8.3)	
Progressive	225 (18.5)		123 (18.8)		102 (18.1)	
Tinnitus^b^	778 (63.9)	—	439 (67.2)	—	339 (60.1)	—
Aural pressure^b^	200 (16.4)	—	114 (17.5)	—	86 (15.2)	—
Dizziness	481 (39.5)	—	282 (43.2)	—	199 (35.3)	—
Balance complaints	306 (25.1)	27 (2.2)	196 (30.0)	13 (2.0)	110 (19.5)	14 (2.5)
Vertigo	120 (9.8)		58 (8.9)		62 (11.0)	
Duration of symptoms (months, median (range))^c^	12 (0‐983)	2 (0.2)	12 (0‐627)	2 (0.3)	13 (0‐983)	—
Koos grade		282 (23.2)		167 (25.6)		115 (20.4)
1	331 (27.2)		129 (19.8)		202 (35.8)	
2	334 (27.4)		187 (28.6)		147 (26.1)	
3	121 (9.9)		73 (11.2)		48 (8.5)	
4	149 (12.2)		97 (14.9)		52 (9.2)	
Median diameter (mm, median (range))						
Intrameatal (parallel to IAC)	8 (0^d^‐16)	302 (24.8)	8 (0^d^‐15)	176 (27.0)	6 (0^d^‐16)	126 (22.3)
Extrameatal						
Perpendicular to petrous bone (parallel to IAC)	4 (0‐24)	293 (24.1)	5 (0‐22)	175 (26.8)	2 (0‐24)	118 (20.9)
Parallel to petrous bone	7 (0‐44)	297 (24.4)	8 (1‐34)	174 (26.6)	6 (0‐44)	123 (21.8)
Aspect on MRI		444 (36.5)		254 (38.9)		190 (33.7)
Homogeneous	532 (43.7)		246 (37.7)		286 (50.7)	
Inhomogeneous	241 (19.8)		153 (23.4)		88 (15.6)	
Cystic	136 (11.2)		85 (13.0)		51 (9.0)	
Non‐cystic	86 (7.1)		54 (8.3)		32 (5.7)	
Unclear	19 (1.6)		14 (2.1)		5 (0.9)	
PTA (dB, median [range])						
250 Hz AC	25 (−5‐110)	587 (48.2)	25 (−5‐110)	335 (51.3)	20 (0‐110)	252 (44.7)
500 Hz AC	25 (0‐120)	587 (48.2)	30 (0‐120)	335 (51.3)	25 (0‐120)	252 (44.7)
1000 Hz AC	40 (0‐120)	587 (48.2)	35 (0‐120)	335 (51.3)	45 (0‐120)	252 (44.7)
2000 Hz AC	55 (−10‐120)	587 (48.2)	55 (0‐120)	335 (51.3)	55 (−10‐120)	252 (44.7)
4000 Hz AC	65 (0‐120)	587 (48.2)	63 (0‐120)	335 (51.3)	65 (5‐120)	252 (44.7)
8000 Hz AC	75 (0‐110)	588 (48.3)	70 (0‐110)	335 (51.3)	75 (0‐110)	253 (44.9)

Abbreviations: AC, air conduction; IAC, internal auditory canal; PTA, pure‐tone audiometry; VS, vestibular schwannoma.

^a^ The number of patients and corresponding percentage is reported, unless stated otherwise in the first column.

^b^ Ipsilateral of VS.

^c^ Duration was set at 0 in case the complaints were absent.

^d^ Intrameatal size can be 0 for intracochlear and exclusively extrameatal VSs.

Potential predictors were entered into a Cox regression model, taking into account the multiple imputed data sets. Akaike's information criterion was used as a selection criterion.^12^ The probability of VS growth at a certain time point can be calculated by using the following formula:

1 − S(t), where S(t) = S_0_(t)^*exp* (β_1_ *x*_1_ + β_2_ *x*_2_ + ... + β_n_ *x*_n_).

In this formula, S(t) is the “survival” of VS, that is the probability of no VS growth. S_0_(t) represents the baseline survival at time t, and β_1_, β_2_ and β_n_ are the regression coefficients of the predictors *x*_1_, *x*_2_ and *x*_n_, respectively, after having been pooled. Baseline survival is defined as the survival for the mean of all covariates in the model and can be transformed into a probability of future growth at the different time points for an individual patient.

For newly diagnosed VS patients assigned to a W&S strategy, predictions within the first five years following diagnosis are of interest to determine timing of the first follow‐up MRI. Predictions at ten years are relevant for a patient's prognosis. Model performance was assessed on calibration using calibration plots for predictions at 1‐5 and 10 years. The model's ability to discriminate between patients with successful or unsuccessful outcomes was estimated using Harrell's C.^13^ Prediction models derived with multivariable regression analyses are known for overfitting. This results in too extreme predictions when the model is applied in new cases. Therefore, it was validated internally using bootstrapping techniques. Five hundred samples were drawn with replacement from the development sample. Bootstrapping techniques provide information on the performance of the model in comparable datasets and generate a shrinkage factor to adjust regression coefficients.^14^ Thereafter, model performance was re‐evaluated.

For development of multivariable prediction models, sample size is often based on the number of events per parameter estimated (EPP). This can be calculated by dividing the number of individuals with or without the outcome (whichever is lower) by the number of parameters to be estimated. We used 22 potential predictors that make up 24 parameters to be estimated (including multiple categories of the variable “Koos grade”), amounting to an EPP of 23 (EPP = 564 “events [no VS growth]” divided by 24 parameters to be estimated). An EPP above 20 is considered to eliminate the estimated bias in regression coefficients and achieve reliable results.^15^, ^16^

A dynamic nomogram was created to easily calculate an individual's risk of VS growth. The nomogram is available via https://vs-model.shinyapps.io/predictVSgrowth, where more data can be entered and corresponding predictions on VS growth can be calculated.

TRIPOD recommends to evaluate netbenefit of prediction models.^16^ Decision curve analysis (DCA) can help to summarise clinical usefulness of prediction models and support in decision making.^17^, ^18^ In a DCA, netbenefit is plotted against threshold probability. In this study, netbenefit represents the proportion of true positives (detected VS growth) in absence of any false positives (ie specificity of 100%).^18^ Threshold probability is defined as the minimum predicted risk of VS growth at which an otolaryngologist or patient would want the first follow‐up MRI. A range of values for the threshold probability is displayed in order to represent a variation in preferences.^19^, ^20^ Interviews with experts in the field of VS revealed a relevant range of risk threshold values of 10% (MRI in 10 patients to detect one case of VS growth and accept 9 false positives, ie unnecessary MRIs) to 30% (MRI in 10 patients to detect 3 cases of growth and accept 7 false positives). DCA was performed for the different time points (1‐5 and 10 years). These can be used to compare the model to a “scan all” (ie the current), and “scan none” strategy and enable one to determine the threshold probability to initiate follow‐up. Furthermore, we calculated the number of MRIs avoided for different threshold probabilities for each of the first five years. Data analysis was performed in R version 3.5.1 (R Foundation for Statistical Computing, Vienna, Austria) using packages “rms” and “rmda".^21^, ^22^

### 2.5 Ethics statement

This study was performed with consent of the local medical ethics committee. The need for informed consent was waived, because of the retrospective nature and size of the study.

## 3 RESULTS

### 3.1 Study population

**Figure 1:**
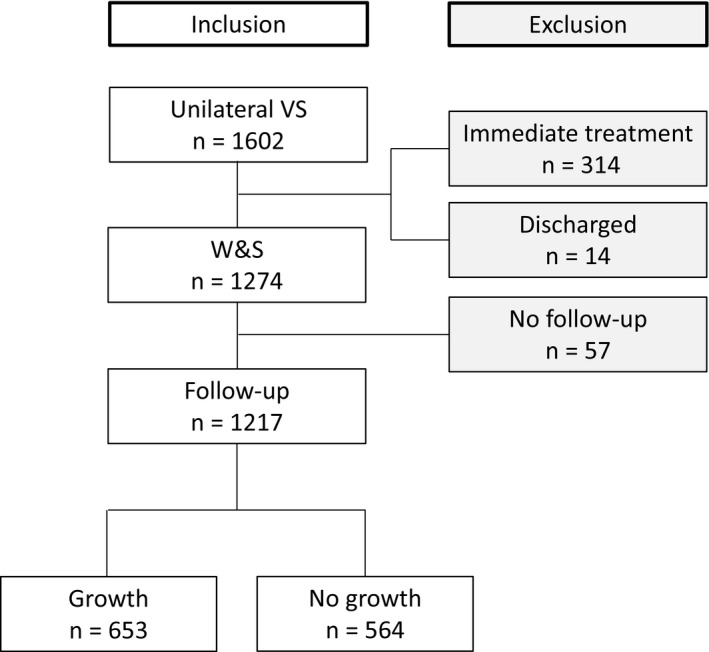
Flow chart displaying patient in‐ and exclusion

We identified 1602 patients with an MRI‐diagnosed unilateral VS. Three hundred and fourteen patients were excluded, because treatment was initiated at time of diagnosis, 239 (14.2%) and 75 (4.7%) were treated with microsurgery and radiation therapy, respectively. Another 14 (0.9%) were discharged from further controls due to patient preference or severe comorbidity. For the remaining 1274 (79.5%) patients, a W&S strategy was initiated. Of these, 1217 had at least one follow‐up MRI available and thus could be included for further study (Figure 1). Of the included VSs, 603 and 614 were located on the right and left side, respectively.

### 3.2 Outcome

#### 3.2.1 VS growth

MRI images were available for review for the majority of patients. Radiologists’ reports were used to determine VS growth in 37.9% of the total number of examinations (n = 3474). In 653 patients (53.7%), VS growth was detected at some point during W&S. Of these, 442 patients (67.7% of patients with a growing VS, or 36.3% of the total study population) also received treatment. Median time to VS growth was 13 months (range: 3‐167), and median censoring time (time to final follow‐up for patients without VS growth) was 44 months (range: 2‐243).

### 3.3 Predictors

Table 1 displays patient and VS characteristics at time of diagnosis. Median age of the included patients was 58.5 years (range 16.5‐88.0), and 51.9% were male. Hearing loss was the most common complaint on the affected ear, followed by tinnitus. Baseline MRI images were available for review for 935 patients (76.8%), for the remaining patients we based VS presence on the radiologist's report. Other baseline MRI characteristics were registered as missing for the latter patients. Most patients presented with a Koos grade 1 (27.2%) or 2 (27.4%) VS. PTA results were available for 630 patients (51.8%).

### 3.4 Multivariable model

After backward selection, the following variables remained in the multivariable model: balance complaints and tinnitus complaints in the affected ear, Koos grading scale, duration of symptoms and the intrameatal diameter (Table 2). After multiplying the regression coefficients with the shrinkage factor (0.97) and updating the intercept, the model's performance was re‐evaluated. The final model's discrimination yielded a Harrell's C of 0.69 (95% confidence interval (CI): 0.67‐0.71), indicating good discrimination.^23^ The model's calibration at the time points of interest was visualised with calibration plots and considered good for predictions at all time points (Figure S1).

**Table 2:** Predictors for VS growth. Using the baseline risk and regression coefficients, a patient's probability of VS growth can be calculated

Predictors	Multivariable analysis	
	Regression coefficient after shrinkage	HR (95% CI)
Balance complaints	0.4360	1.57 (1.31‐1.88)
Tinnitus	0.2998	1.36 (1.15‐1.61)
Koos grade 1	reference	reference
Koos grade 2	0.0250	1.03 (0.80‐1.31)
Koos grade 3	0.4240	1.55 (1.16‐2.06)
Koos grade 4	0.7542	2.18 (1.60‐2.96)
Time since onset of symptoms (months)	−0.0046	0.83 (0.77‐0.88)^a^
Intrameatal diameter (mm)	0.1237	1.67 (1.42‐1.95)^a^

Note

Baseline survival is defined as the survival for the mean of all covariates in the model. Growth probability for a new patient can be calculated using the formula: 1 − S(t), where S(t) = S_base_^exp(lp), and S_base_ is the baseline survival at the time point of interest, and lp is the centred linear predictor. The baseline survival for time points 1‐5 and 10 y are as follows: S_base12_ = 0.7707905, S_base24_ = 0.6238292, S_base36_ = 0.5362379, S_base48_ = 0.4889781, S_base60_ = 0.4496488, S_base120_ = 0.3244630] The linear predictor can be manually calculated as: *lp* = 0.4360386 * (Balance complaints − 0.2670501) + 0.024917 * (Koos grade 2 − 0.3393591) + 0.423974 * (Koos grade 3 − 0.1322925) + 0.7541666 * (Koos grade 4 − 0.1930978) + −0.0045785 * (Time since onset − 43.5937962) + 0.1237266 * (Intrameatal diameter − 7.3360723) + 0.2998087 * (Tinnitus − 0.6392769). See also: https://vs-model.shinyapps.io/predictVSgrowth.

Abbreviation: HR, hazard ratio.

^a^ Interquartile range hazard ratio (interquartile range).

Patients with balance complaints (hazard ratio (HR) 1.57 (95% CI: 1.31‐1.88)) and tinnitus complaints in the affected ear (HR 1.36 (95% CI: 1.15‐1.61)), a higher Koos grade at time of diagnosis (HRs of 1.03 (95% CI: 0.80‐1.31), 1.55 (95% CI: 1.16‐2.06) and 2.18 (95% CI: 1.60‐2.96) for Koos grade 2, 3 and 4, respectively), short duration of symptoms (interquartile range (IQR) HR 0.83 (95% CI: 0.77‐0.88)) and a larger intrameatal diameter at time of diagnosis (IQR HR 1.67 (95% CI: 1.42‐1.96)) have a higher probability of future VS growth (Table 2).

#### 3.4.1 Example

**Figure 2:**
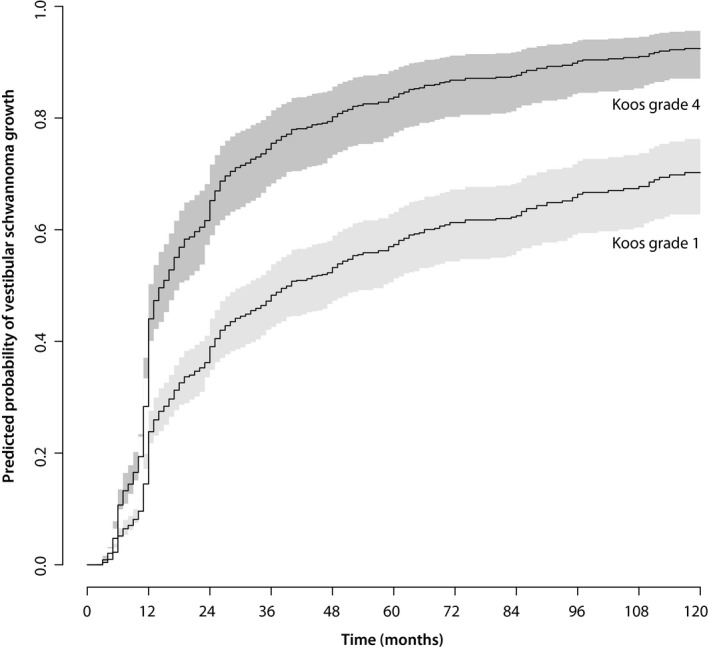
Predicted probabilities for a patient whose complaints started 12 mo ago, who has tinnitus but no balance problems, whose VS has an intrameatal diameter of 8 mm and is classified as Koos grade 1 (light grey) or Koos grade 4 (dark grey) at diagnosis

Using the proposed multivariable model for a patient whose complaints started 12 months ago, who has tinnitus but no balance problems, whose VS has an intrameatal diameter of 8 mm, and is classified as Koos grade 1, would have a probability of VS growth of 38% (95% CI: 32%‐43%) two years following diagnosis. For five years following diagnosis, this probability increases to 55% (95% CI: 48%‐61%) (Figure 2). A patient with the same characteristics, with the exception of having a Koos grade 4 VS rather than a Koos 1 grade at time of diagnosis would have a probability of future VS growth of 64% (95% CI: 56%‐70%) and 82% (95% CI: 75%‐87%) at 2, and 5 years following diagnosis, respectively (Figure 2). More variations can be entered online to calculate predictions at different time points (https://vs-model.shinyapps.io/predictVSgrowth).

### 3.5 Decision curves

**Figure 3:**
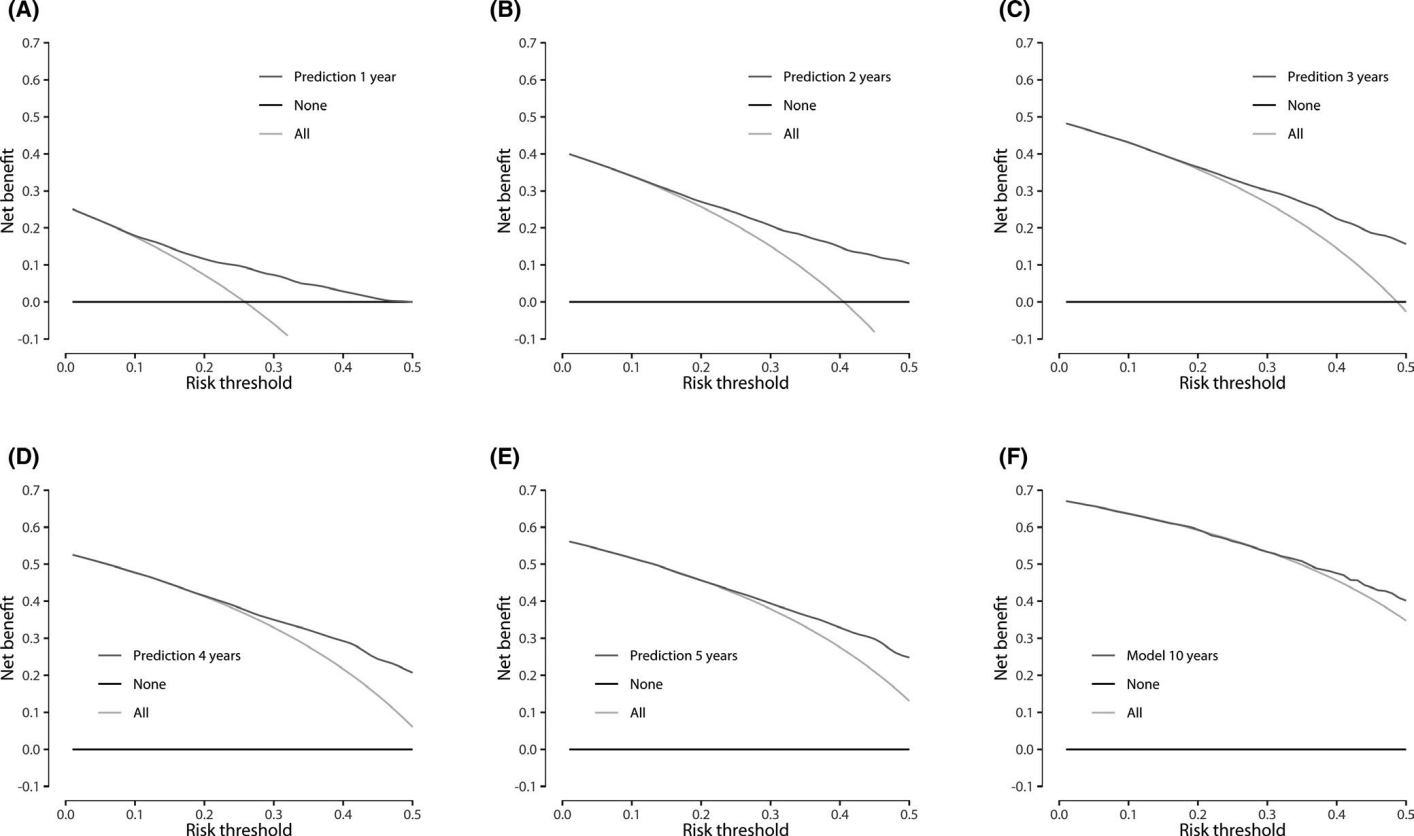
Netbenefit curves for time points 1‐5 and 10 y. The *x*‐axis represents the risk threshold and the *y*‐axis the net benefit. Netbenefit represents the proportion of true positives (detected VS growth) in absence of any false positives (ie specificity of 100%).^18^ The black line represents a strategy in which no MRIs are acquired; the netbenefit is 0. The light grey line represents the current strategy, in which all patients have undergone an MRI. The dark grey line represents the prediction model

**Figure 4:**
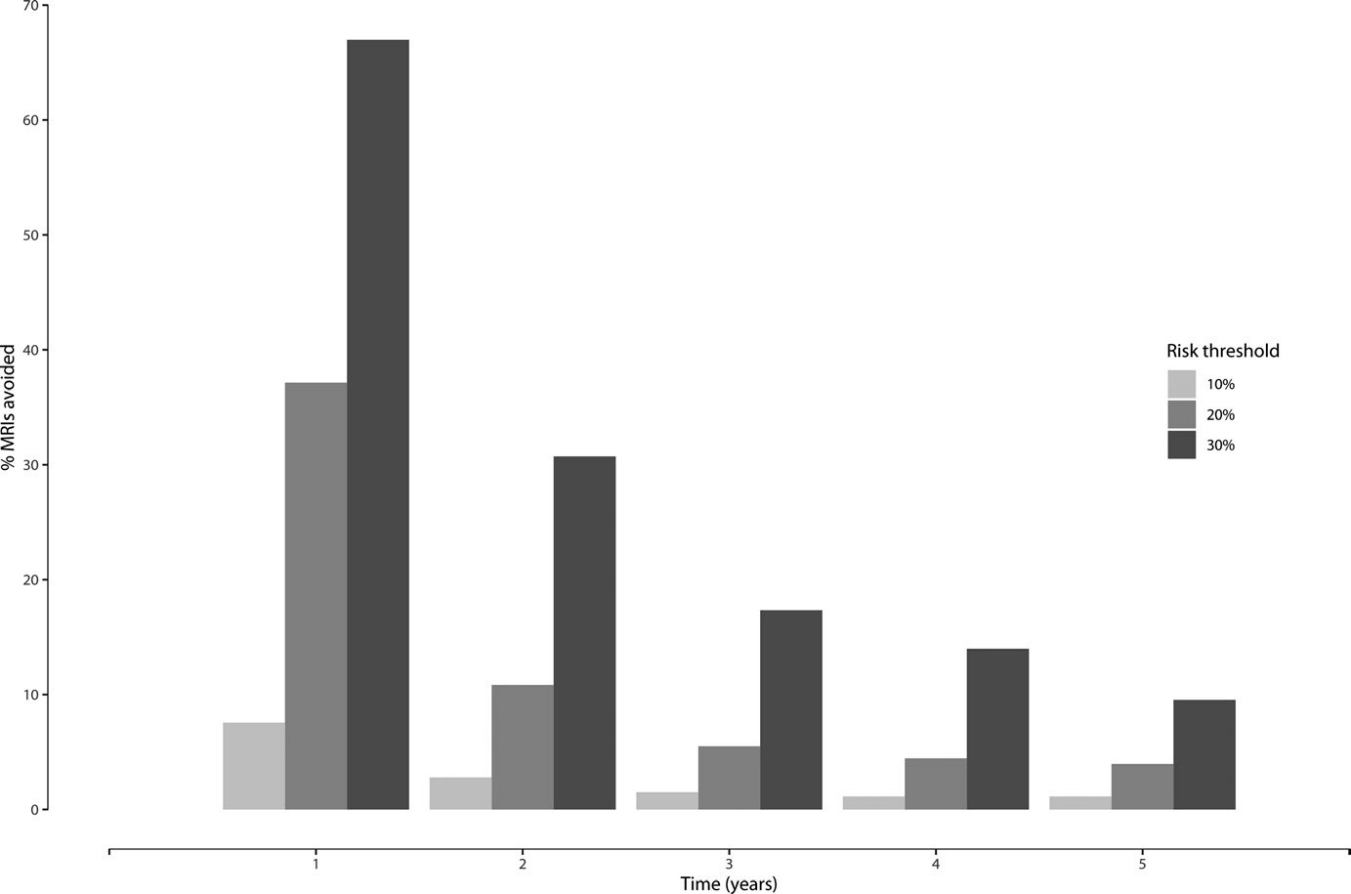
MRIs avoided for different risk thresholds and time points. The *x*‐axis displays different time points. The *y*‐axis displays the proportion of MRIs avoided. Light grey = risk threshold of 10%. Grey = risk threshold of 20%. Dark grey = risk threshold of 30%

Figure 3 displays the netbenefit curves of the model for predictions at the different time points. The strategy with the highest netbenefit regarding the detection of VS growth at a specific threshold probability is clinically most useful. At risk thresholds >12%, 15%, 21%, 23%, 25% and 35% for years 1‐5, and 10 years, respectively, the developed model has a higher netbenefit compared to scanning all patients (Figure 3). Figure 4 displays the percentage of MRIs avoided for risk thresholds of 10%, 20% and 30% for each of the first five years.

## 4 DISCUSSION

We developed a multivariable time‐to‐event model predicting VS growth in newly diagnosed patients assigned to a W&S strategy. Our results show that patients with balance complaints and tinnitus complaints in the affected ear, a higher Koos grade, short duration of symptoms and a larger intrameatal diameter at time of diagnosis have a higher probability of VS growth. This prediction model may be helpful in the development of new W&S strategies and contributes to individualised care for patients diagnosed with VS. Individual patient data can be entered online (https://vs-model.shinyapps.io/predictVSgrowth) to calculate a patient's probability of VS growth.

Several authors have tried to identify features of VS that might predict future growth, growth rate and/or treatment. Most, however, looked at VS subgroups (eg VSs limited to the internal auditory canal^24^, ^25^ or non‐cystic VSs^26^, ^27^) used small sample sizes,^3^, ^24^, ^28^, ^29^, ^30^, ^31^, ^32^, ^33^ used volume measurements to assess growth^30^ or included growth rate in the first year of follow‐up in their analyses.^32^ Based on findings of studies on the topic, including the current study, we might state that age and sex are no strong predictors for VS growth.^4^, ^24^, ^29^, ^30^, ^31^, ^32^, ^33^, ^34^, ^35^ Multiple authors did find an association between VS growth or treatment and VS size at time of diagnosis,^4^, ^24^, ^26^, ^36^, ^37^ balance complaints^4^, ^26^, ^35^ or extension in the CPA.^26^, ^33^, ^38^, ^39^

The largest comparable study comprising 564 patients was performed by Hunter et al^4^ They evaluated risk factors for VS growth and found similar results, with larger initial VS diameter and disequilibrium complaints being identified as significant predictors (with increased HRs for both).^4^ Age, sex, asymmetrical hearing loss and vertigo were not identified as significant predictors.^4^ Tinnitus, however, was not selected in their model, whereas it was in the current study. This difference in findings could be explained by the fact that we, unlike Hunter et al, linked presence of symptoms to the affected ear.^4^

We have collected data of a large cohort of patients with unilateral VS. To our knowledge, this is the largest study reporting on a complete cohort of VS patients in a W&S strategy with a relatively long duration of follow‐up (mean 41 months). Predictors included in the model consist of presenting symptoms, baseline MRI parameters and PTA results, which can easily be obtained in every otolaryngology practice. By comparing each MRI to the baseline MRI (instead of the previous MRI), we were able to identify slow growing lesions.

Some potential limitations should also be discussed. First, PTA results were available for a slight majority of patients. From 2003 onwards, PTA results were digitally available. Thus, data from earlier days are missing at random (MAR). Second, MRI images were assessed by one person [MH]. Although inter‐ and intra‐observer reliability is high for VS measurements, it is not 100%.^40^ Also, the aspect of VS (inhomogeneous and cystic) on MRI is a partially subjective parameter. We, however, decided not to add a second reader as in daily clinical practice the outcome will also be assessed by a single person.

Third, we had to rely on radiologists’ reports rather than MRI images in a large minority of cases (37.9%). Although assessment by radiologists from another institution might have been slightly different from our measurement method, we assumed that the presence of growth (yes/no) was properly assessed in these cases. In case of suspected growth, patients were usually referred to our clinic and MRI images could be assessed. For examinations of which we had both a report and measurements available, we used our measurements for analyses. We were able to compare our findings to the radiologist's report in these cases, and agreement was reached in 79%.

Regional otolaryngologists might have reported less often about stable VSs compared to growing VSs, since the latter are referred to our centre for further management. This might have resulted in an overestimation of VS growth. The proportion of patients with VS growth varies in literature, which is partially explained by abovementioned differences in study methods and follow‐up. The proportion of VS growth found in our study (53.7%) was comparable to a study by Artz et al.^26^ Kirchmann et al,^25^ who studied intracanalicular VSs (Koos grade 1) observed growth in 37% of patients, which is similar to our Koos 1 patients (38%). In the study by Hunter et al,^4^ growth was detected in 40.8% of patients. However, the fact that 36.3% of our patients was eventually treated is comparable to their data.^4^

Fourth, VS growth was defined as a ≥2 mm increase in diameter, while slice thickness was larger in the MRI examinations from the earliest study period. This might have resulted in an underreporting of VS growth in these earlier MRIs. Finally, we measured VS size in two directions, which might have led to missed growth in another direction.

The model was developed and internally validated in a Dutch population. External validation is necessary prior to its clinical use. Subsequently, the proposed multivariable model can be used in the consulting room to assess an individual patient's probability of having future VS growth. These findings can, next to patient counselling, also be used to establish a more individualised W&S strategy for patients. Increasing the interval between subsequent MRIs is relatively safe in selected patients, because potential growth can still be identified at a later time.

The data from this study enable further study of new W&S strategies. As mentioned previously, the range of threshold probabilities deemed relevant by specialists was 10%‐30%. When making predictions for 1‐5 years following diagnosis, the model performs better than the current strategy with threshold probabilities within range preferred by the experts, that is the thresholds were >12%, 15%, 21%, 23% and 25%, respectively. After 10 years, the model performs similar to a “scan all” strategy for the preferred threshold range of 10%‐30%, and for a threshold of >35%, the model has a higher net benefit than the current strategy.

Of all VSs that grew, <5% and 1% did so after 7 and 10 years, respectively. Given these data, we would at least suggest termination of follow‐up after 10 years for non‐growing VSs.

Future studies might reveal which changes in symptoms should prompt patients to visit their clinician. In case the model's performance could be further improved, it might even be possible to safely omit selected patients from further controls. It is difficult to assess the impact of missed VS growth and subsequent delayed treatment on clinical outcomes, especially since our data show that growth ≥2 mm does not necessarily lead to treatment. Stereotactic radiosurgery is usually not performed in VSs exceeding 3 cm, so delayed detection of growth beyond this size will result in a more invasive treatment, that is microsurgery.^41^ So far, long‐term quality of life seems comparable for both treatment strategies, although results according to VS size are unknown.^42^

Abandoning all monitoring will initially result in the greatest cost reduction. However, based on current knowledge, long‐term cost‐effectiveness (including quality of life) and functional outcomes of the latter strategy are difficult to assess.

Further exploration of new W&S strategies, including their cost‐effectiveness, is needed to reach an optimal W&S schedule.

## 5 CONCLUSION

Patients with balance and tinnitus complaints, a higher Koos grade, short duration of symptoms and a larger intrameatal diameter at time of diagnosis have a higher probability of future VS growth following diagnosis. Clinicians may use these variables to determine which recently diagnosed patients in a W&S strategy should be monitored more carefully.

## CONFLICT OF INTEREST

None to report.

## AUTHOR CONTRIBUTIONS

Design and conception by MH, HK, SS, MR. MH, HK, JM, SS contributed to data collection. MR, GH, MH were involved in data analyses. All authors contributed to writing and revising the manuscript.

## ETHICAL CONSIDERATIONS

This study was performed with consent of the local medical ethics committee. The need for informed consent was waived, because of the retrospective nature and size of the study.

## ROLE OF THE FUNDING SOURCE

The funding source did not have any role in study design; in the collection, analysis and interpretation of data; in the writing of the report; and in the decision to submit the paper for publication.

## Supporting information

Figure S1

## Data Availability

The data sets used and/or analysed during the current study are available from the corresponding author on reasonable request and approval by the local privacy officer.
