# Supplementary figures and images for: Development of a model to predict vestibular schwannoma growth: An opportunity to introduce new wait and scan strategies
Source: Clin Otolaryngol. 2020 Nov 6;46(1):273–83. doi: 10.1111/coa.13661 (PMC7821120; doi:10.1111/coa.13661)

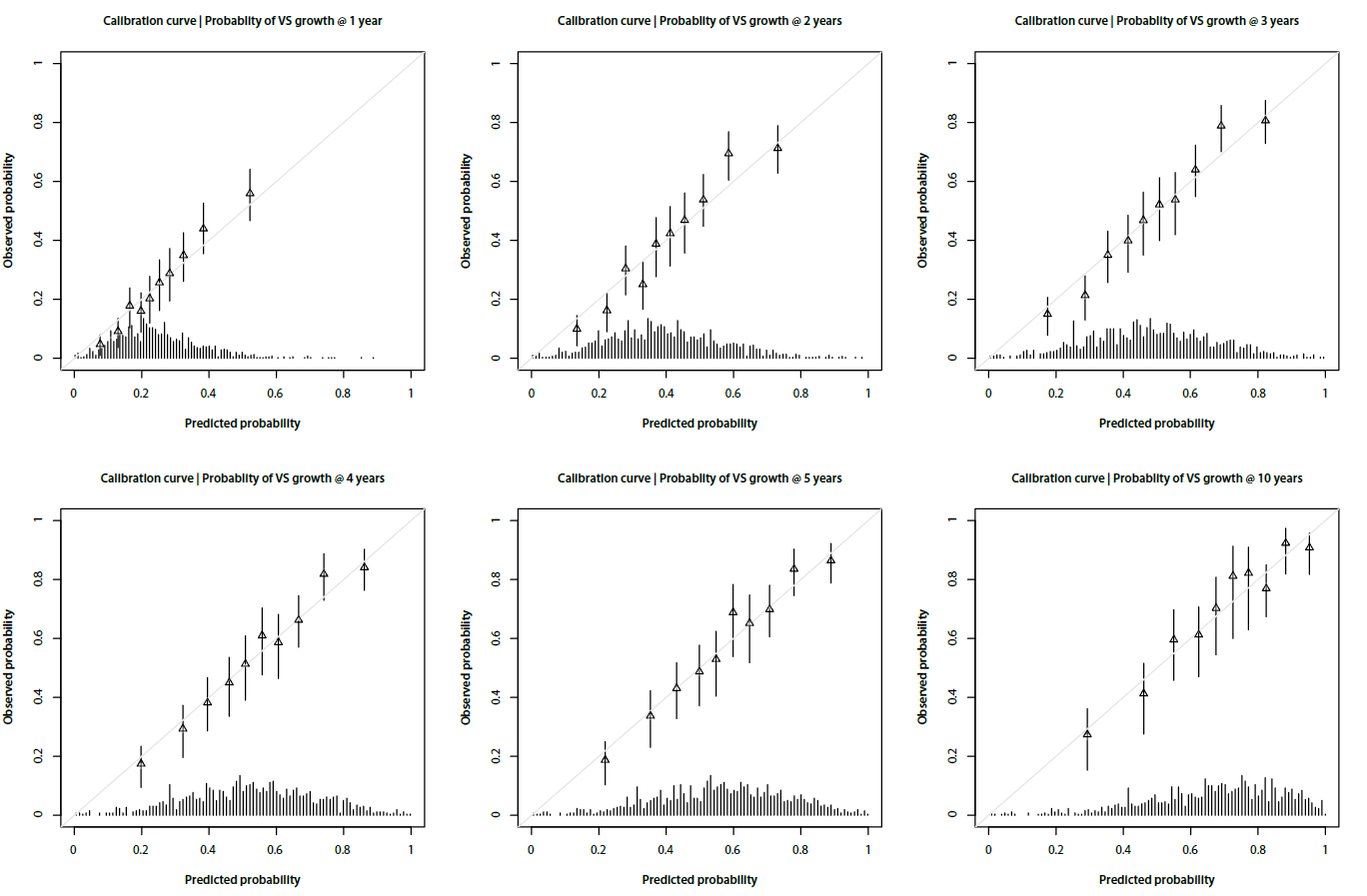

Supplement: Supplementary file 1 — Figure S1 [file COA-46-273-s001.png]
